# Functional Impairments and Work-Related Outcomes in Breast Cancer Survivors: A Systematic Review

**DOI:** 10.1007/s10926-017-9736-8

**Published:** 2017-10-30

**Authors:** Rimke Bijker, Saskia F. A. Duijts, Sherzel N. Smith, Renée de Wildt-Liesveld, Johannes R. Anema, Barbara J. Regeer

**Affiliations:** 1 Athena Institute, VU University Amsterdam, Amsterdam, The Netherlands; 2 Division of Psychosocial Research and Epidemiology, The Netherlands Cancer Institute, Amsterdam, The Netherlands; 3 Department of Public and Occupational Health, VU University Medical Center, Van der Boechorststraat 7 – C573, 1081 BT Amsterdam, The Netherlands; 4 Research Center for Insurance Medicine, AMC-UMCG-UWV-VUmc, Amsterdam, The Netherlands

**Keywords:** Breast cancer, Functional impairments, Return to work, Occupational health services

## Abstract

*Purpose* Work participation after breast cancer treatment is generally negatively affected. Occupational health professionals might improve work-related outcomes by bridging the gap between sick-listed employees’ levels of functioning and work demands. To aid them in this task, this review explored the association between functional impairments and work-related outcomes in breast cancer survivors. *Methods* Publications from January 2000–March 2016 were identified through five online databases (i.e. Pubmed, EMBASE, PsycINFO, CINAHL and the Cochrane Library). Quantitative and qualitative studies were included if they focused on functional impairments and work-related outcomes in breast cancer survivors. Two reviewers independently selected studies, extracted data and performed quality assessment. *Results* The search identified 998 studies, of which 20 studies met eligibility criteria. Impairments in physical functioning negatively affected return to work (RTW) and work ability in quantitative and qualitative studies. Studies measuring cognitive functioning with tests found no association with work-related outcomes, whereas the results of studies using self-reported measures were ambiguous. Social functioning was less commonly investigated and findings differed across work-related outcomes. Emotional functioning was not associated with work-related outcomes in quantitative studies, while in qualitative studies feelings such as insecurity were described as influencing RTW. *Conclusions* Functional impairments can severely hamper work participation in breast cancer survivors. This provides important opportunities for occupational health professionals to enhance RTW in breast cancer survivors, such as adequately addressing illness perceptions and work expectations. Ongoing research is warranted to aid occupational health professionals in providing effective vocational guidance and improve work-related outcomes in breast cancer survivors.

## Introduction

The incidence of breast cancer is increasing globally [1], and although early diagnosis and better treatment options have improved survival, participation in society after breast cancer is generally negatively affected [2]. Many women are not able to return to work (RTW) or experience diminished work outcomes, such as increases in sick leave and lowered work ability [2–4]. This puts an economic burden on society, since about 70% of new breast cancer cases occur in women of working age [5]. Moreover, being able to work is of great importance on an individual level as well, as work contributes to a sense of normalcy [6], financial security [7–9], and improved quality of life [10, 11].

Evidence from systematic reviews related to cancer and work reveals that factors associated with RTW in cancer survivors include, among others, age, socio-economic status, disease stage, type of treatment and treatment-related symptoms [12, 13]. In addition, it has been suggested that successful RTW is influenced by the extent to which the cancer survivor’s level of functioning meets the demands at work [14]. For example, breast cancer surgery is associated with lymphedema [15], which in turn can impair arm function [16] and consequently may affect specific physical tasks in different work environments. Also, chemotherapy and its related side effects may induce cognitive impairment [17, 18], which may influence the ability to concentrate while at work. As such, successful RTW might be enhanced by interventions that include vocational support, to help overcome the discrepancy between the level of functioning of breast cancer survivors and the demands of work.

The potential of vocational support is further emphasized in a recent meta-analysis that summarized the effects of various RTW interventions in cancer survivors [19]. Among the evaluated interventions were monodisciplinary interventions (including physical, psycho-educational and medical interventions) and multidisciplinary interventions (interventions that combined aspects of monodisciplinary interventions with vocational components). None of the monodisciplinary interventions showed a beneficial effect on RTW. By contrast, moderate quality evidence was found that RTW was positively influenced by multidisciplinary interventions which combined physical and psycho-educational components with vocational components. These findings underline the importance of providing vocational guidance and occupational health services for those who are returning to work.

As in several other high-income countries, in the Netherlands, employers are required to offer occupational health services [20]. These services are generally provided by occupational health professionals. Part of their responsibilities includes the facilitation of vocational rehabilitation. Internationally, occupational health professionals are required to have a profound base of general medical knowledge and to be commonly familiar with the workplace and work tasks [21, 22]. Therefore, they are in an ideal position to provide vocational guidance. More specifically, they can aid sick-listed employees by helping them increase their level of functioning to meet work demands, or by adjusting the work environment so that employees can work despite functional impairments.

To offer proper vocational guidance, it is necessary that occupational health professionals have knowledge regarding the relation between the level of functioning of sick-listed employees and the ability to resume work. To our knowledge, this relationship with respect to breast cancer survivors has not previously been addressed by systematic reviews. Yet, providing an overview of this topic is especially relevant as the growing number of working age breast cancer survivors implies that these women will constitute an increasing proportion of the occupational health professionals’ tasks. Therefore, the aim of this review was to explore the association between functional impairments and work-related outcomes in breast cancer survivors.

## Methods

### Search Strategy

A systematic search was performed in the databases PubMed, EMBASE, PsycINFO, CINAHL and in the Cochrane Library, restricted to studies published from January 2000 until March 2016. Studies were identified using search syntaxes based on the PubMed strategy, which uses a combination of MeSH terms and free text terms that were related to breast cancer, functional impairments and employment. Subsequently, the search syntax was adapted per database, including different or additional search terms where necessary (Appendices 1, 2, 3, 4 and 5). Functional impairment was defined as limitations due to a condition or its treatment that prevent people from carrying out certain functions in their daily life. Breast cancer survivor refers to women who have been diagnosed with breast cancer, regardless of breast cancer stage, time since diagnosis and type of treatment. Studies published in English were eligible for inclusion if they evaluated functional impairments in relation to work-related outcomes in breast cancer survivors with an employment contract at time of diagnosis. Both original quantitative and qualitative studies of which the study populations comprised working age adults were included. Studies were excluded if the majority of the study population had a condition or cancer type other than breast cancer, if there was no mention of functional impairments (for instance if only symptoms were evaluated) or if the work-related outcomes were focused on economic consequences only, such as a loss of income.

### Study Selection

**Fig. 1:**
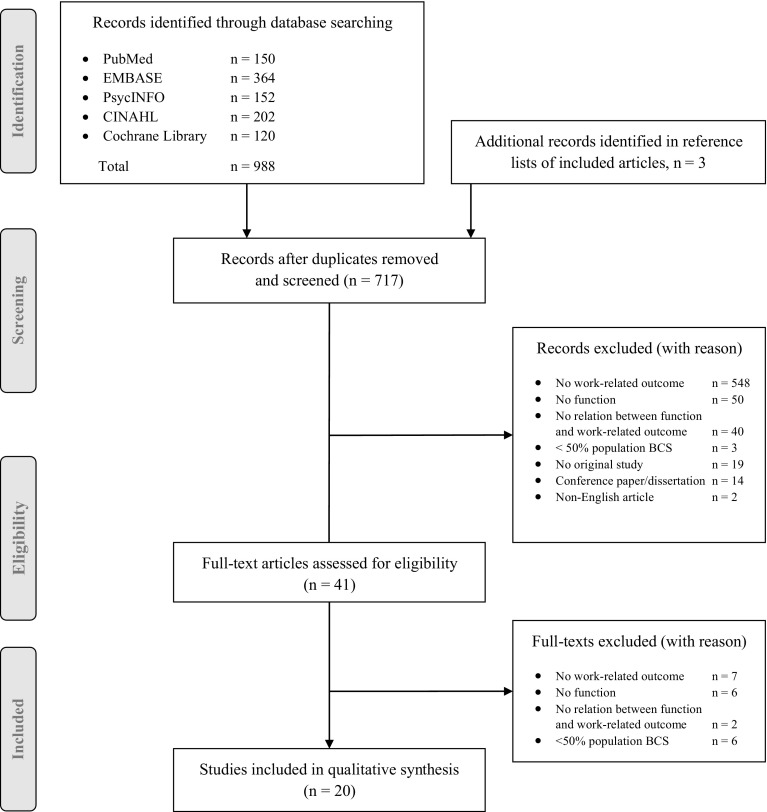
Study selection

Study selection was performed in three steps. First, the search results were screened by title and abstract. Second, full-text articles were retrieved to assess if they met the inclusion criteria. Third, a manual search of reference lists of included articles was conducted to identify further relevant studies. The first two steps were independently performed by two authors (RB and SS). In case there was no consensus regarding the eligibility of the articles, a third author (SD) decided if the article should be included in the review. For articles that were excluded, reasons for not including them are documented in Fig. 1.

### Data Extraction and Synthesis

A data extraction form was created to record relevant study details. One author (RB) extracted data on country, study design, population (e.g., number of participants, age, breast cancer stage and received breast cancer treatment), measures of functional impairments (e.g., physical, cognitive, social functioning), work-related outcomes (e.g., sick leave, return to work, work performance and work retention) and main findings of the study. We derived our measures of functional impairments from the European Organization for Research and Treatment of Cancer Quality of Life Questionnaire (EORTC QLQ) since this tool has been widely used in cancer research. We carefully assessed the EORTC QLQ and decided to use its scales regarding functioning as predefined categories to extract and group information on functional impairments. To adequately reflect the findings grouped under each category we later defined the categories as general and role functioning (i.e. overall functioning and the ability to perform common tasks at home and work), physical functioning (i.e. physical ability in general, physical work ability and shoulder functioning), cognitive functioning (i.e. cognitive ability in general, mental work ability, memory, concentration, focusing, processing, executive functioning and multitasking), social functioning (i.e. pursuing social activities), and emotional functioning (i.e. emotional functioning in general, emotional response to condition and ability to deal with stress). The extracted data were checked by another author (SS) and in cases of disagreement, a third author (SD) decided which data needed to be reported. Subsequently, the study characteristics and the results of the included studies were summarized by functional impairment and grouped according to work-related outcome if applicable.

### Quality Assessment

The methodological quality of the studies was scored independently by two authors (RB, RWL), using quality assessment checklist for prognostic studies, case-control studies and qualitative studies that were derived from the Critical Appraisal Skills Program (CASP) [23] and for cross-sectional studies we used checklists derived from the Strengthening the Reporting of Observational Studies in Epidemiology (STROBE) [24]. Each of these checklists contains items related to the clarity of the research objective, appropriateness of the research design, appropriateness of the sampling strategy, description of the method of analysis, and clarity of the data description. Inconsistencies in scoring were discussed until agreement was reached by two researchers (RB, RWL). In case studies did not score positively on each of the items on the checklists, we described in what aspect (i.e. on which items) they were lacking quality.

## Results

### Study Characteristics

In total, 998 studies were identified from the systematic search and three additional relevant studies were found in the manual reference list search of included articles. After removing duplicates, and exclusion based on title and abstract, 41 full-text articles were retrieved for full-text screening. Of these, 21 studies were excluded because they did not meet the selection criteria (Fig. 1). An overview of the main characteristics of the remaining 20 studies and their findings is provided in Tables 1 and 2. In short, 11 studies had a quantitative design [25–35] and nine had a qualitative design [7, 36–43]. The majority of the studies was conducted in the United States (n = 7) [26, 27, 30, 33, 36, 38, 43] and Europe (Netherlands, n = 3 [25, 37, 41]; United Kingdom, n = 3 [7, 28, 39]; Sweden, n = 3 [31, 32, 40]; joint cohort Nordic countries, n = 1 [29]), two studies were conducted in Canada [34, 35] and one in Malaysia [42]. Seven studies were of prospective nature, with follow-up periods ranging from 3 months to 4 years [25, 28, 31–34] and 13 studies had a cross-sectional design [7, 26, 27, 29, 30, 35–43]. One study was reported in two articles, with the first comprising the baseline results [35] and the other reporting the findings at long-term follow-up [34]. Four studies reported on short-term outcomes, occurring in the first year after breast cancer diagnosis [28, 31, 35, 40], while 14 studies reported on long-term outcomes [7, 25–27, 29, 30, 32–34, 36, 38, 39, 41, 43]. Two qualitative studies did not clearly define how much time had elapsed between breast cancer diagnosis and the problems participants disclosed [37, 42]. Study sample sizes ranged from n = 44 to n = 1111 breast cancer survivors in the quantitative studies and from n = 10 to n = 74 survivors in the qualitative studies. None of the studies included male breast cancer survivors in their study sample. In two studies, the populations consisted of a mixed cancer group, in which seven out of ten participants (70%) [37] and 219 out of 431 participants (51%) [29] were treated for breast cancer. In only two studies, the results regarding the relation between impairment of functioning and work were compared to a control group [26, 30].

**Table 1:** Characteristics and study findings of quantitative studies on functional impairments and work-related outcomes in breast cancer survivors

Author, year [Ref.] Country	Design, (follow-up)	Time since diagnosis	Participants n, *age*	Breast cancer stage	Treatment	Functional impairments	Measurement	Outcome	Findings
Balak, 2008 [25] Netherlands	Longitudinal, prospective cohort (24 months)	> 1 year	72 BCS, *mean age 49 years* (*SD 7*)	0, I, II	Surgery, chemotherapy, radiotherapy, combination treatment	Shoulder functioning (range of motion)	Medical records	Duration until RTW	Shoulder function impairment was associated with prolonged duration until partial RTW (HR 0.48; 95% CI 0.23–0.98) No significant associations were found between shoulder function impairment and duration until full RTW
Calvio, 2010 [26] United States	Cross-sectional with controls	> 1 year	122 BCS, *mean age 45 years* (*SD 10*) *113 controls, mean age 39 years* (*SD 12*)	I, II, III	Surgery, chemotherapy, radiotherapy, combination treatment	Cognitive functioning	CSC, internet-based performance tests	Work limitations	Self-reported measures of cognitive limitations were associated with work output limitations in BCS (memory: β = 0.29; p < 0.05 and executive function β = 0.26; p < 0.05), but not in controls Performance-based measures of cognitive impairment were associated with work output limitations in controls (executive function: β = − 0.36; p < 0.01 and attention: β = 0.33; p < 0.05) but not in BCS
Cleeland, 2014 [27] United States	Cross-sectional	Ns	280 BCS, *mean age 57 years* (*range 29–94*), of whom 58 employed BCS, *age ns*	Newly diagnosed locally recurrent or metastatic breast cancer	Chemotherapy, hormonal therapy, targeted therapy, neo-adjuvant therapy	Functional status (i.e., ability to perform common tasks)	ALS	Work productivity (work time missed, impairment while working, overall work impairment)	Better functional status was associated with a decrease in work time missed (estimate of − 1.4%; p = 0.0182), impairment while working (estimate of − 1.2%; p = 0.0102), and overall work impairment (estimate of − 0.9%; p = 0.0383)
Cooper, 2013 [28] United Kingdom	Longitudinal, prospective (12 months)	> 1 year^b^	89 BCS, *mean age 49 years* (*SD 7*)	Non-metastatic	Surgery, chemotherapy, radiotherapy, hormonal therapy, combination treatment	Role, physical, cognitive, social and emotional functioning	EORTC QLQ-C30	Duration until RTW	No significant associations were found for any of the functioning scales and duration until RTW
Gudbergsson, 2008 [29] Denmark, Finland, Iceland, Norway	Cross-sectional	> 1 year	431 CS (of whom 219 BCS) with or without work changers, *mean age 51 years* (*SD 8*) *and 50 years* (*SD 10*), *resp*	I	Surgery, chemotherapy, radiotherapy, hormonal therapy, radiotherapy	Physical and mental work ability (due to cancer)	WAI	Work changes due to cancer (change of employer, occupation, work tasks, unemployment or pensioning), current work ability	Reduced physical work ability (OR 2.58; 95% CI 1.18–5.56; p = 0.02) associated with an increased chance of work changes No significant associations were found for mental work ability and work changes Reduced physical work ability (β = − 0.407; p < 0.001) and reduced mental ability (β = − 0.466; p < 0.001) were associated with a reduction of current work ability
Hansen, 2008 [30] United States	Cross-sectional with controls	> 1 year^b^	100 BCS, *mean age 50 years* (*SD 9*) 103 non-cancer comparisons, *mean age 40 years* (*SD 11*)	I, II, III	Surgery, chemotherapy, radiotherapy, other (ns)	Cognitive functioning	CSC	Work limitations	No significant associations were found between cognitive limitations and work limitations in BCS No significant differences were found between the BCS and the control group
Hedayati, 2012 [31] Sweden	Longitudinal (3 months)	≤ 1 year	44 BCS, who returned to work or were still on sick leave, *mean age 54 years* (*SD 6*) *and 53 years* (*SD 6*), *resp*	I, II	Surgery, chemotherapy hormonal therapy, no adjuvant therapy	Neurocognitive functioning (attention, memory, response speed, processing speed), role, physical, cognitive, social and emotional functioning	CSI, EORTC QLQ BR-23	RTW	Better role functioning (OR 0.94; 95% CI 0.91–0.96; p < 0.001), physical functioning (OR 0.83; 95% CI 0.74–0.90; p < 0.001), and social functioning (OR 0.96; 95% CI 0.93–0.99; p < 0.001) were associated with an increased chance to RTW No significant associations were found between cognitive limitations, cognitive functioning and emotional functioning, and RTW
Lundh, 2014 [32] Sweden	Longitudinal, population-based (4 years)	> 1 year	463 BCS, *median age 54 years* (*range 25–62*), of whom 441 women without distant metastasis were included in the analysis	No metastases or recurrence	Surgery, chemotherapy, radiotherapy, hormonal therapy, antibody therapy	Emotional functioning	EORTC QLQ-C30 and QLQ-BR23	Sickness absence	No significant associations were found between emotional functioning and sickness absence at 2nd or 3rd year post-diagnosis
Oberst, 2010 [33] United States	Longitudinal (18 months)^a^	≤ 1 year > 1 year^c^	447 BCS, *mean age 50 years* (*SD 8*)	In-situ, local, regional, distant/unknown	Surgery, chemotherapy, radiotherapy, combination treatment, no treatment	Physical and cognitive functioning	Questionnaire completion through telephone interview	Employed, left workforce, average workday duration	Physical disability was associated with a lower likelihood of being employed at 12 months (77.8 vs. 90.0%; p < 0.01) and 18 months (75.5 vs. 96.4%; p < 0.01) Physical disability was associated with a higher likelihood of leaving the workforce at 12 months (11.9 vs. 4.2%; p < 0.01) and 18 months (11.7 vs. 0.6%; p < 0.01) Cognitive disability was associated with a lower likelihood of being employed at 18 months (77.8 vs. 90.0%; p < 01), but not at 12 months Cognitive disability was associated with a higher likelihood of leaving the workforce at 12 months (14.0 vs. 4.2%; p < 0.01) and 18 months (12.8 vs. 0.7%; p < 0.01) No significant associations were found between those with or without physical or cognitive disability and worked hours a day
Quinlan, 2009 [35] Canada	Cross sectional	≤ 1 year	278 BCS, with and without loss of productivity, *mean age 53* (*SD 8*) *and 50* (*SD 8*), *resp*	I, II, III	Surgery, chemotherapy, radiotherapy, hormonal therapy	Range of motion	Clinical assessment	Loss of productivity	ROM limitation was associated with decreased productivity (increased loss of productivity) (OR 2.55; 95% CI 1.20–5.43; p = 0.015)
Quinlan, 2011 [34] Canada	Longitudinal (36 months)	> 1 year	372 BCS^d^, *age* > *18 years, mean age ns*	I, II, III	Surgery, chemotherapy, radiotherapy, hormonal therapy	Range of motion	Clinical assessment	Loss of productivity	ROM limitation was associated with decreased productivity (increased loss of productivity) at 6–12 months (OR 3.12; 95% CI 1.45–6.69; p = 0.003) and 30–36 months (OR 4.08; 95% CI 1.09–15.34; p = 0.037) post-surgery

*BCS* breast cancer survivor, *ns* not specified, *CSC* cognitive symptom checklists, *ALS* Activity Level Scale, *EORTC QLQ-C30* European Organization for Research and Treatment of Cancer Quality of Life Questionnaire, *WAI* Work Ability Index, *CSI* Cognitive Stability Index, *QLQ-BR23* Breast Cancer specific Quality of Life Questionnaire, *ROM* range of arm motion, *RTW* return to work, *SD* standard deviation, *IQR* interquartile range, *resp*. respectively, *HR* hazard ratio, *CI* confidence interval, *p* probability value, *OR* odds ratio

^a^Results are based on univariate analysis

^b^Time since diagnosis for majority of participants;

^c^Outcomes reported for multiple time points

^d^Employed or seeking employment at the beginning of the study

**Table 2:** Characteristics and study findings of qualitative studies on functional impairments and work-related outcomes in breast cancer survivors

Author, year [Ref.] Country	Design	Time since diagnosis	N participants, *age*	Breast cancer stage	Treatment	Functional impairments	Measurement	Outcome(s)	Findings
Boykoff, 2009 [36] United States	Cross-sectional	> 1 year	74 BCS, *age 30–89 years*	Ns	Surgery, chemotherapy, radiotherapy, hormonal therapy	Cognitive and emotional functioning	Interviews, FGD	Job performance	Forgetfulness, memory loss, loss of words and concentration problems make it harder to do a job. Cognitive problems can lead to frustration and stress, which make it harder to maintain or find a job
Groeneveld, 2013 [37] Netherlands	Cross-sectional	Ns	10 CS, *mean age 56 years* (*SD 6*), of whom 7 BCS, *mean age 52 years*	Ns	Surgery, chemotherapy, radiotherapy	Cognitive and emotional functioning	Interviews	Work performance	Problems with concentration can lead to making more mistakes while working. Some respondents are stressed during early RTW and start crying at work for seemingly pointless reasons
Hinman, 2008 [38] United States	Cross-sectional	> 1 year^a^	31 BCS, *mean age 57 years* (*range 38–79*)^b^	Ns	Surgery	Physical functioning	Comment section in survey	Work re-entry	Limitations in using the arm hampers work task such as carrying a heavy load and thereby inhibits RTW
Kennedy, 2007 [7] United Kingdom	Cross-sectional	> 1 year^a^	29 CS, *mean age 53 years*, of whom 24 BCS, *mean age 52 years* (range 36–66)	Ns	Surgery, chemotherapy, radiotherapy and/or hormonal therapy	Cognitive and emotional functioning	Interviews, FGD	Work ability, sickness absence	Some respondents reported that they were able to return to work, function effectively and perform as they had previously. Others revealed how it was initially difficult to cope and concentrate; they worried about their reduced capability at work. For some respondents, pressure, emotional demands, insecurity and worries about appearance make it difficult to manage work
Munir, 2010 [39] United Kingdom	Cross-sectional	> 1 year	13 BCS, *mean age 49 years* (*range 32–57*)	Primary breast cancer diagnosis	Surgery, chemotherapy, radiotherapy, hormonal therapy	Cognitive and emotional functioning	FGD	Work ability	Chemotherapy-induced cognitive impairment, such as problems with memory, concentration, decision-making and multitasking, affected women’s views and experiences towards returning to work and their confidence in their work ability
Nilsson, 2013 [40] Sweden	Cross-sectional	≤ 1 year^a^	23 BCS, *mean age 53 years* (*range 37–62*)	No metastasis	Surgery, chemotherapy, radiotherapy, hormonal therapy	General functioning	FGD	Work-related issues	The women’s general functioning, including emotional consequences of treatment, influenced their decision to take actions whether to work or to be absent after diagnosis, during treatment, or after treatment. Cognitive side effects led to less work capacity than desired or to less efficiency at work
Tamminga, 2012 [41] Netherlands	Cross-sectional	> 1 year^a^	12 BCS, *mean age 42 years* (*SD 7*)	Primary breast cancer diagnosis with or without metastasis	Surgery, chemotherapy, radiotherapy, hormonal therapy	Physical and cognitive functioning	Interviews	RTW	Having difficulties with mobility of joint functions, having difficulties with attention functions, retrieval of memory, pace of thought and higher-level cognitive functions were perceived as impairments to RTW
Tan, 2012 [42] Malaysia	Cross-sectional	Ns	40 BCS, *mean age in RTW group 43 years* (*SD 10*), *mean age in non-RTW group 49 years* (*SD 5*)	I, II, III	Surgery, radiotherapy, chemotherapy, hormonal therapy	Physical, cognitive and emotional functioning	FGD	RTW	Physical limitations (such as not being able to walk long distances or carry weight) caused by disease hampered RTW. Other reported impairments were related to cognitive function, such as forgetfulness and slowness in thinking. Changing emotional states like worrying and frustrations leads to low frustration tolerance and poor decision making on RTW
Von Ah, 2013 [43] United States	Cross-sectional	> 1 year	22 BCS who reported cognitive limitations, *mean age 56 years* (*SD 9.7, range 40–74*)	I–III	Chemotherapy, radiotherapy, hormonal therapy	Cognitive and emotional functioning	Interviews	Work ability	Both short-term and long-term memory problems, decreased speed of processing, limited attention, concentration, language and executive functioning were reported to impact work ability. Concerns regarding cognitive abilities and lack of confidence made some respondent leave their prior job

*BCS* breast cancer survivors, *CS* cancer survivors, *SD* standard deviation, *RTW* return to work, *FGD* focus group discussion, *Ns* not specified

^a^Time since diagnosis for majority of participants

^b^48.8% of women worked prior to their surgery

### Quality Assessment

Overall, we agreed that the methodological quality of the studies was high. For 11 studies, all items in the quality assessment were scored positively [27–32, 34, 37, 40, 41, 43]. In the remaining articles reporting on quantitative studies there may have been confounding [33], some measurement bias [25], or there was a lack of clarity regarding participant sampling [26, 35]. In one article there was also insufficient discussion of potential bias, generalizability and the interpretation of the results [35]. Concerns about articles reporting on qualitative studies were mainly related to adequately addressing ethical issues [7, 36, 38] and considering the relationship between researcher and participants [7, 38, 39, 42]. Furthermore, one of these articles also scored negatively on the appropriateness of the research design and the method of data analysis [38]. Taking into consideration the assessed quality of the studies, we decided not to deploy a weight difference when describing the results.

### Quantitative Studies

A total of 11 studies reported quantitative results regarding one or more domains of functioning [25–35]. Three studies described general functioning [27, 28, 31], seven studies described physical functioning [25, 28, 29, 31, 33–35], six studies described cognitive functioning [26, 28–31, 33], two studies described social functioning in general [28, 31], and finally, three studies described emotional functioning in general [28, 31, 32]. These domains were evaluated by means of medical assessment [25, 34, 35], telephone interviews [33], neuropsychological performance tests [26], or questionnaires, such as the Cognitive Symptom Checklist [26, 30], Activity Level Scale [27], Work Ability Index (which covers physical and cognitive work ability) [29], Cognitive Stability Index [31], or EORTC QLQ-C30 [28, 32] and Breast Cancer-Specific Quality of Life Questionnaire (QLQ-BR23) [31, 32]. The domains of functioning were investigated in relation to work ability [26, 27, 29, 30, 34, 35], RTW [28, 31], duration until RTW [25, 28], employment status [29, 33], sickness absence [27, 32] and working hours [33].

#### General and Role Functioning

Functional status in general and role functioning were investigated in relation to various work-related outcomes. The findings indicated that better functional status was associated with less sickness absence, and higher work productivity [27]. Better role functioning was associated with a slightly increased chance to RTW [31], but not with the duration until RTW [28].

#### Physical Functioning

Generally, problems with physical functioning were associated with negative work outcomes. For instance, a higher proportion of breast cancer survivors with physical disabilities was not employed or had left the workforce at 12 and 18 months after diagnosis [33]. In addition, reduced physical work ability led to more than a twofold increase in work changes and less overall work ability [29]. More specifically, problems with shoulder functioning were reported to impact RTW and work ability after RTW. For example, limited range of motion was associated with a loss of productivity [35], which was still apparent 2.5–3 years after surgery [34]. Furthermore, shoulder functioning impairment prolonged sick leave duration until partial RTW, but not until full RTW [25]. Interestingly, general physical functioning was not associated with duration until RTW [28] or working hours [33].

#### Cognitive Functioning

Cognitive functioning was evaluated by means of performance-based test [26, 31] and self-reported measures [26, 28–30, 33]. Breast cancer survivors with low scores on neuropsychological performance tests did not differ from those who had high scores with regard to RTW [31] and work output [26]. Findings from self-reported measures were somewhat inconsistent. Breast cancer survivors with a higher level of subjective cognitive impairment were more likely to be unemployed, to have left the workforce [33], or have lower work output [26]. However, other findings indicated that subjective cognitive functioning was not associated with work-related outcomes, such as duration until RTW [28], work productivity [30], working hours [33], and work changes [29].

#### Social and Emotional Functioning

Less commonly investigated in relation to work-related outcomes were the domains of social and emotional functioning. Better social functioning was associated with higher RTW rates [31], but not with the duration until RTW [28]. With respect to emotional functioning, none of the findings showed significant associations with work-related outcomes in breast cancer survivors [28, 31, 32].

### Qualitative Studies

A total of nine studies reported qualitative results regarding one or more domains of functioning [7, 36–43]. One study described general functioning [40], three studies described physical functioning [38, 41, 42], seven studies described cognitive functioning [7, 36, 37, 39–41, 43], and seven studies described emotional functioning [7, 36, 37, 39, 40, 42, 43]. Study participants were asked about the various domains through interviews [7, 36, 37, 41, 43], focus group discussions [7, 36, 39, 40, 42] or a comment section in a survey [38]. The domains of functioning were mainly described in relation to RTW [38–42], and work ability [7, 36, 37, 39, 40, 43].

#### General Functioning

Impaired functioning in general was described as the driver of decisions on going to work or taking sick leave immediately following diagnosis, during treatment and in the phase thereafter [40].

#### Physical Functioning

Problems with mobility and executing physical tasks, such as carrying and walking, were reported to hamper RTW [38, 41, 42]. This became clear from studies in which women related their physical impairments to specific tasks at work. For example, a participant in the study by Tan et al. [42] explained: “I am physically tired; I was not able to walk long distance, and not able to monitor work because I noticed I was breathless during walking or going up a flight of stair.” In some cases, the decision not to resume a job is made by others than the breast cancer survivor, which was explained by one woman in a study, in which women were interviewed who had undergone a mastectomy: “I was the assistant manager of a convenience store and did a lot of heavy lifting and stacking. They would not take me back after the surgery” [38].

#### Cognitive Functioning

The findings showed that work-related outcomes were greatly impacted by cognitive impairments, including problems with concentration, attention, memory, pace of thought, multitasking, executive functioning, speed of processing and decision-making. These impairments were perceived to be related to the process of returning to work [39, 41, 42], as well as to problems with work ability by occupationally active breast cancer survivors [7, 36, 37, 39, 40, 43]. Impairments in cognitive functioning commonly became apparent beyond RTW, as was explained by a 51-year old senior receptionist in a study on chemotherapy-induced cognitive problems: “It was when I went back to work I noticed, I felt as though I’d had a lobotomy” [39]. Especially when numerous cognitive functions are required for completing a job task, this was described as leading to problems when working: “It makes my job a lot harder, because as a teacher you have to do everything all at once. So, when I leave at the end of the day, I am spent, when before I was energetic. And it’s not a physical spent; it is a mental spent that I didn’t used to have” [36]. Fortunately, the negative impact of cognitive impairment was also reported to diminish as time passed by, which was discussed by women, who had undergone breast surgery, in a focus group study: “I had been on sick leave for a month when I realized that I could not concentrate, but now I work just as before” [40].

#### Emotional Functioning

Breast cancer diagnosis and treatment were reported to affect emotional functioning, which influenced choices on RTW. For instance, returning to work was described as a source of stress, at times leading women to tear up [7, 37]. Furthermore, low-spiritedness, fears, worries, frustrations and insecurity about appearances made it challenging for some breast cancer survivors to resume employment [7, 40, 42]. One woman elaborated on her insecurity at work after getting a breast prosthesis: “I had to lean down to do anything on the bottom, lower shelf or even for bags to pack them, I was like this [covered her chest] all the time, holding it together… every minute of my working day you’re thinking of it” [7]. Cognitive impairment resulting from treatment was frequently cited as an additional reason for insecurity and frustration [36, 39, 43]. These problems in turn were explained to change the experience of work as it used to be, which for instance made an office manager retire early: “With this memory thing, I was very frustrated at work and so I thought that I can’t go on like this. It was a chore now going to work than a joy. I just assessed the situation and said that it’s not worth it” [36].

## Discussion

In this systematic review, we explored the association between functional impairments and work-related outcomes in breast cancer survivors. The findings show that overall, better functional status was related to more favourable work-related outcomes. Impairments in physical functioning were consistently described as negatively impacting RTW and work ability in both quantitative and qualitative studies. With regards to cognitive functioning, the findings were inconsistent across studies. Studies measuring cognitive functioning with neuro-psychological performance-based tests found no association with work-related outcomes, whereas the results of studies using self-reported measures of cognitive function were ambiguous. In qualitative studies, however, cognitive impairments were frequently reported as hampering RTW and diminishing work ability. Social functioning was less commonly investigated and findings differed across work-related outcomes. Emotional functioning was not associated with work-related outcomes in quantitative studies, while in qualitative studies, feelings such as stress, fear, worries, frustration, insecurity and low-spiritedness were described as influencing decisions on RTW.

### Interpretation of Findings

The findings show that physical functioning was univocally related to RTW and work ability, whereas findings for other domains of functioning were not as straightforward. This might partly be because in scientific literature, the concept of work disability is primarily focused on physical aspects of functioning, and to a lesser extent on cognitive, social and emotional aspects [44]. This might be reflective of what happens in practice. Indeed, occupational health physicians evaluating disability in cancer survivors have reported to rely mainly on a biomedical approach, while subjective complaints of psychosocial functioning, which are harder to assess, take a less prominent position [45].

As previously reported for cancer survivors in general [46], our review confirmed a difference between self-reported and performance-based measures of cognitive functioning. Studies have shown little correlation between these measures in cancer survivors [47, 48]. It has been suggested that breast cancer survivors might perform better at tests because they are aware of their limitations and try to overcome them in a test setting [26]. Furthermore, it is possible that performance-based tests are not sensitive in picking up impairments in cognitive functioning which are required for specific tasks at work. Hence, occupational health professionals should be cautious in generalising test results to cognitive functioning needed at work. Instead, to facilitate favourable work-related outcomes, it seems expedient to interpret cognitive functioning in light of each individual’s daily work activities.

Our review showed that, in various studies, breast cancer survivors reported emotional functioning to negatively impact work participation. According to a study among Japanese and Dutch participants, emotional responses elicited by breast cancer are stronger than those in individuals with other chronic diseases, such as asthma and diabetes [49, 50]. Furthermore, other findings have indicated that suppressing emotional responses to breast cancer might be related to emotional impairment [51]. Research suggests that social support and the ability to disclose feelings are pivotal in coping with emotional issues caused by breast cancer [51, 52]. However, evidence shows that there is a high unmet need for support with these issues among breast cancer survivors [53]. Taken together, these findings allude to the importance of providing the information and support to help women cope with their condition and lessen emotional struggle [51, 52]. Subsequently, this might improve overall functioning and facilitate work participation.

Interestingly, in qualitative studies participants consistently reported that functional impairments negatively affected RTW and work ability, while findings were divergent across quantitative studies. This might be attributable to how breast cancer survivors perceive their health condition, and the difference in manifestation of these perceptions in qualitative and quantitative studies. Firstly, impairments can subjectively be experienced as debilitating by breast cancer survivors, even though they might be too subtle to objectively determine. A possible explanation for this difference is that individuals commonly overestimate their pre-disease level of functioning and consequently set unrealistic rehabilitation goals [54]. Secondly, according to Leventhal, individuals form a set of beliefs about their disease and the consequences thereof, based on their personal experiences, medical knowledge, and environmental input [55], which may mediate or exacerbate outcomes in the period following illness. As shown by a recent review, illness perceptions of breast cancer survivors indeed appear to be linked to various important health and behavioural outcomes [56]. For instance, having a strong belief that diagnosis and treatment lead to serious symptoms or problems with activities of daily life has been associated with poorer mental and physical health [57]. Likewise, work-related outcomes may be affected by illness perception as well. This is illustrated by a review which reports that believing one’s illness is long-lasting and has serious consequences for health and daily life is more often seen in non-working individuals than in those with more favourable illness perceptions [58].

### Strengths and Limitations

The main strength of this review is that, rather than discussing determinants in general of RTW of breast cancer survivors, we focussed specifically on functional impairments in relation to work outcomes. By distilling a more homogenous set of findings, our review provides a unique perspective which can provide practical guidance to those in the field of occupational medicine. Specifically, our findings give direction to how occupational health professionals can support breast cancer survivors in returning to work and retain them on the work floor. Another strength is that, by including both quantitative and qualitative studies, we revealed the potential importance of perceptions regarding work participation after breast cancer.

An important limitation to the study is that findings are difficult to compare between countries, due to major differences in social security systems [59]. For instance, in countries such as Canada and the Netherlands it is possible to work under therapeutic conditions, that is, to resume part-time work and gradually increase work activities and working hours over the course of multiple years while receiving partial disability benefits [60, 61]. In other countries, however, disability benefits are only granted in case of more severe work incapacity, though at the same time, employees are at risk of termination of their employment contract [59]. As a result, RTW cannot be interpreted similarly across countries. In correspondence to this, the heterogeneity in measurement of work-related outcomes and social security systems in which these outcomes are embedded prevents the possibility of pooling quantitative results and conducting a meta-analysis, which would provide stronger evidence.

### Implications for Practice and Research

Occupational health professionals should be aware that experienced problems in functioning that influence work participation might not be objectively measurable. That is, illness perceptions of breast cancer survivors play an essential role in RTW, and research has shown a discrepancy between the illness perceptions of employees and occupational health physicians [62]. Further, breast cancer survivors should receive an overview of potential side effects of treatment and possible consequences to their functional status, specifically in relation to future work resumption. By increasing medical knowledge and addressing unfavourable illness perceptions, occupational health professionals can facilitate a smoother RTW process. Additionally, helping breast cancer survivors to revise unrealistic expectations might contribute to less emotional problems such as distress and frustration [54].

Our findings put forward important directions for future research. First, we found a wide variety of work-related outcomes, which implies the need for a common framework to assess work participation. Second, there is a lack of literature on important work-related outcomes after cancer, such as changes in work activities and working hours. Research on these outcomes is warranted, since they may be desirable end points if work resumption at the pre-disease level is an unrealistic goal. Finally, the importance of perceptions regarding work participation after breast cancer should be further investigated in research.

## Conclusions

Our findings indicate that functional impairments can severely hamper work participation in breast cancer survivors. Notwithstanding, there might be important opportunities for occupational health professionals to enhance RTW and work retention in breast cancer survivors. Specifically, opportunities exist in adequately addressing illness perceptions and work expectations. Ongoing research is needed to aid occupational health professionals in providing effective vocational guidance and improve work-related outcomes in breast cancer survivors.

## Appendix 1: PubMed Search Strategy

**Table:**

Search	Query	Items found
#5	Search **(#4)** Filters: **Publication date from 2000**/**01**/**01**	150
#4	#1 AND #2 AND #3	194
#3	“Recovery of Function“[Mesh] OR “Disability Evaluation“[Mesh] OR “Sickness Impact Profile“[Mesh] OR “Physical Fitness“[Mesh] OR “Movement“[Mesh] OR impairment*[tiab] OR disabilit*[tiab] OR Capabilit*[tiab] OR capacit*[tiab] OR impair*[tiab] OR function*[tiab] OR dysfunction*[tiab] OR limitation*[tiab] OR restriction*[tiab] OR physical fitness[tiab] OR movement*[tiab] OR mobilit*[tiab] OR EORTC QLQ[tiab] OR Functional Assessment of Cancer Therapy[tiab] OR SF-36[tiab] OR functional abilit*[tiab] OR functional capa*[tiab]	4,518,304
#2	“Convalescence“[Mesh] OR “Absenteeism“[Mesh] OR “Sick Leave“[Mesh] OR “Return to Work“[Mesh] OR “Work Performance“[Mesh] OR “Unemployment“[Mesh] OR “Retirement“[Mesh] OR “Work Capacity Evaluation“[Mesh] OR (“Efficiency“[Mesh] AND (work*[tiab] OR job*[tiab])) OR convalescen*[tiab] OR absenteeism[tiab] OR work absence*[tiab] OR disability absence*[tiab] OR sickness absence*[tiab] OR sick day*[tiab] OR illness day*[tiab] OR work day loss*[tiab] OR work time loss*[tiab] OR medical leave*[tiab] OR sick leave*[tiab] OR sickness leave*[tiab] OR disability leave*[tiab] OR presenteeism[tiab] OR sickness presence[tiab] OR return-to-work[tiab] OR back- to-work[tiab] OR reintegration[tiab] OR reemployment[tiab] OR job reentry[tiab] OR work productivit*[tiab] OR work function*[tiab] OR work participation[tiab] OR work performance*[tiab] OR performance at work[tiab] OR employment status[tiab] OR work status[tiab] OR unemployment[tiab] OR unemployed[tiab] OR work abilit*[tiab] OR workability[tiab] OR work disabilit*[tiab] OR work inabilit*[tiab] OR work capacit*[tiab] OR work incapacity[tiab] OR work capabilit*[tiab] OR work incapabilit*[tiab] OR work inhibition*[tiab] OR work function*[tiab] OR job function*[tiab] OR work participation[tiab] OR work performanc*[tiab] OR job performanc*[tiab] OR vocational performanc*[tiab] OR performance at work[tiab] OR work productivit*[tiab] OR work efficien*[tiab] OR job efficien*[tiab] OR work retention[tiab] OR work sustainability[tiab] OR retirement*[tiab] OR working hour*[tiab] OR work hour*[tiab] OR work task*[tiab] OR working task*[tiab] OR task at work[tiab] OR tasks at work[tiab] OR job task*[tiab]	93,643
#1	“Breast Neoplasms“[Mesh] OR ((“Breast“[Mesh] OR breast[tiab]) AND (“Neoplasms“[Mesh] OR neoplas*[tiab] OR cancer*[tiab] OR carcin*[tiab] OR tumour*[tiab] OR tumor*[tiab] OR metasta*[tiab] OR malig*[tiab]))	319,566

## **Appendix 2**: Embase Search Strategy

**Table:**

Search	Query	Items found
#5	#4 AND (2000:py OR 2001:py OR 2002:py OR 2003:py OR 2004:py OR 2005:py OR 2006:py OR 2007:py OR 2008:py OR 2009:py OR 2010:py OR 2011:py OR 2012:py OR 2013:py OR 2014:py OR 2015:py OR 2016:py)	364
#4	#1 AND #2 AND #3	402
#3	‘disability’/exp OR ‘functional status assessment’/exp OR ‘Sickness Impact Profile’/exp OR ‘functional status’/exp OR ‘fitness’/exp OR ‘movement (physiology)’/exp OR impairment*:ab,ti OR disabilit*:ab,ti OR capabilit*:ab,ti OR capacit*:ab,ti OR impair*:ab,ti OR function*:ab,ti OR dysfunction*:ab,ti OR limitation*:ab,ti OR restriction*:ab,ti OR ‘physical fitness’:ab,ti OR movement*:ab,ti OR mobilit*:ab,ti OR ‘EORTC QLQ’:ab,ti OR ‘Functional Assessment of Cancer Therapy’:ab,ti OR ‘SF-36’:ab,ti OR ‘functional abilit*’:ab,ti OR ‘functional capa*’:ab,ti	5.354.001
#2	‘absenteeism’/exp OR ‘job performance’/exp OR ‘presenteeism’/exp OR (‘productivity’/exp AND (work:ab,ti OR job:ab,ti)) OR ‘return to work’/exp OR ‘work capacity’/exp OR ‘medical leave’/exp OR ‘employment status’/exp OR ‘unemployment’/exp OR ‘retirement’/exp OR convalescence:ab,ti OR absenteeism:ab,ti OR ‘work absence*’:ab,ti OR ‘disability absence*’:ab,ti OR ‘sickness absence*’:ab,ti OR ‘sick day*’:ab,ti OR ‘illness day*’:ab,ti OR ‘work day loss*’:ab,ti OR ‘work time loss*’:ab,ti OR ‘medical leave*’:ab,ti OR ‘sick leave*’:ab,ti OR ‘sickness leave*’:ab,ti OR ‘disability leave*’:ab,ti OR ‘presenteeism’:ab,ti OR ‘sickness presence’:ab,ti OR ‘return-to-work’:ab,ti OR ‘back- to-work’:ab,ti OR reintegration:ab,ti OR reemployment:ab,ti OR ‘job reentry’:ab,ti OR ‘employment status’:ab,ti OR ‘work status’:ab,ti OR unemployment:ab,ti OR unemployed:ab,ti OR ‘work abilit*’:ab,ti OR workabilit*:ab,ti OR ‘work disabilit*’:ab,ti OR ‘work inabilit*’:ab,ti OR ‘work capacit*’:ab,ti OR ‘work incapacit*’:ab,ti OR ‘work capabilit*’:ab,ti OR ‘work incapabilit*’:ab,ti OR ‘work inhibition*’:ab,ti OR ‘work function*’:ab,ti OR ‘job function*’:ab,ti OR ‘work participation’:ab,ti OR ‘work performanc*’:ab,ti OR ‘job performanc*’:ab,ti OR ‘vocational performance’:ab,ti OR ‘performance at work’:ab,ti OR ‘work productivit*’:ab,ti OR ‘work efficien*’:ab,ti OR ‘job efficien*’:ab,ti OR ‘work retention’:ab,ti OR ‘work sustainability’:ab,ti OR retirement:ab,ti OR ‘working hour*’:ab,ti OR ‘work hour*’:ab,ti OR ‘work task*’:ab,ti OR ‘working task*’:ab,ti OR ‘task at work’:ab,ti OR ‘tasks at work’:ab,ti OR ‘job task*’:ab,ti	125.134
#1	‘breast cancer’/exp OR ‘breast cancer’:ab,ti OR (‘breast’/exp OR breast:ab,ti AND (‘neoplasm’/exp OR ‘neoplasm’:ab,ti OR cancer*:ab,ti OR neoplasm*:ab,ti OR carcin*:ab,ti OR tumor*:ab,ti OR tumour*:ab,ti OR metastas*:ab,ti))	485.200

## **Appendix 3**: PsycInfo Search Strategy

**Table:**

Search	Query	Items found
#5	Limiters—Publication Year: 2000–2016	152
#4	S1 AND S2 AND S3	165
#3	MM “Disability Evaluation” OR MM “Physical Fitness” OR TI impairment* OR AB impairment* OR TI disabilit* OR AB disabilit* OR TI capabilit* OR AB capabilit* OR TI capacit* OR AB capacit* OR TI impair* OR AB impair* OR TI function* OR AB function* OR TI dysfunction* OR AB dysfunction* OR TI limitation* OR AB limitation* OR TI restriction* OR AB restriction* OR TI “physical fitness” OR AB “physical fitness” OR TI movement* OR AB movement* OR TI mobilit* OR AB mobilit* OR TI “EORTC QLQ” OR AB “EORTC QLQ” OR TI “Functional Assessment of Cancer Therapy” OR AB “Functional Assessment of Cancer Therapy” OR TI “SF-36” OR AB “SF-36” OR TI “functional abilit*” OR AB “functional abilit*” OR TI “functional capa*” OR AB “functional capa*”	940,188
#2	MM “Employee Absenteeism” OR MM “Employee Leave Benefits” OR MM “Disability Evaluation” OR MM “Reemployment” OR ((MM reintegration OR TI reintegration OR AB reintegration) AND (TI work OR AB work OR TI job OR AB job)) OR MM “Job Performance” OR MM “Employee Efficiency” OR MM “Employee Productivity” OR MM “Employment Status” OR MM “Self-Employment” OR MM “Unemployment” OR MM “Retirement” OR TI convalescence OR AB convalescence OR TI absenteeism* OR AB absenteeism* OR TI “work absence*” OR AB “work absence*” OR TI “disability absence*” OR AB “disability absence*” OR TI “sickness absence*” OR AB “sickness absence*” OR TI “sick day*” OR AB “sick day*” OR TI “illness day*” OR AB “illness day*” OR TI “work day loss*” OR AB “work day loss* OR TI “work time loss*” OR AB “work time loss*” OR TI “medical leave*” OR AB “medical leave*” OR TI “sick leave*” OR AB “sick leave*” OR TI “sickness leave*” OR AB “sickness leave*” OR TI “disability leave*” OR AB “disability leave*” OR TI presenteeism OR AB presenteeism OR TI “sickness presence” OR AB “sickness presence” OR TI “return to work” OR AB “return to work” OR TI “back to work” OR AB “back to work” OR TI reintegration or AB reintegration OR TI “Reemployment” OR AB “Reemployment” TI “job reentry” OR AB “job reentry” OR TI “work productivit*” OR AB “work productivit*” OR TI “work function*” OR AB “work function*” OR TI “job function*” OR AB “job function*” OR ((TI work OR AB work OR TI job OR AB job OR TI vocational OR AB vocational) AND (TI “Performanc*” OR TI “Efficien*” OR TI “Productiv*” OR “capacity* OR TI disabilit* OR AB disabilit*)) OR TI “employment status” OR AB “employment status” OR TI “work status” OR AB “work status” OR TI “unemployment” OR AB “unemployment” OR TI “unemployed” OR AB “unemployed” OR TI “work abilit*” OR AB “work abilit*” OR TI “workabilit*” OR AB workabilit*” OR TI “work disabilit*” OR AB work disabilit*” OR TI “work inabilit*” OR AB work inabilit*” OR TI “work participation” OR AB “work participation” OR TI “work retention” OR AB “work retention” OR TI “work sustainability” OR AB”work sustainability” OR TI “retirement” OR AB “retirement” OR TI “working hour*”OR AB “working hour*” OR TI “work hour*”OR AB “work hour*” OR TI “work task*” OR AB “work task” OR TI “working task*” OR AB “working task” OR TI “task at work” OR AB “task at work” OR TI “tasks at work” OR AB “tasks at work” OR TI “job task*” OR AB “job task*”	135,480
#1	MM “Breast Neoplasms” OR ((MM “Neoplasms” OR MM “Metastasis” OR TI “neoplas*” OR AB “neoplas*” OR TI “metastas*” OR AB “metastas*” OR TI “cancer*” OR AB “cancer*” OR TI “carcin*” OR AB “carcin*” OR TI “tumour*” OR AB “tumour*” OR TI “tumor*” OR AB “tumor*” OR TI “malig*” OR AB “malig*”) AND (MM “Breast” OR TI “breast” OR AB “breast”))	11,055

## **Appendix 4**: CINAHL Search Strategy

**Table:**

Search	Query	Items found
#5	Limiters—Published Date: 20000101–20151231	202
#4	S1 AND S2 AND S3	230
#3	MM “Functional Status” OR MM “Functional Assessment+” OR MM “Disability Evaluation+” OR MM “Work Capacity Evaluation” OR MM “Sickness Impact Profile” OR MM “Physical Fitness+” OR MM “Movement+” OR TI impairment* OR AB impairment* OR TI disabilit* OR AB disabilit* OR TI capabilit* OR AB capabilit* OR TI capacit* OR AB capacit* OR TI impair* OR AB impair* OR TI function* OR AB function* OR TI dysfunction* OR AB dysfunction* OR TI limitation* OR AB limitation* OR TI restriction* OR AB restriction* OR TI “physical fitness” OR AB “physical fitness” OR TI movement* OR AB movement* OR TI mobilit* OR AB mobilit* OR TI “EORTC QLQ” OR AB “EORTC QLQ” OR TI “Functional Assessment of Cancer Therapy” OR AB “Functional Assessment of Cancer Therapy” OR TI “SF-36” OR AB “SF-36” OR TI “functional abilit*” OR AB “functional abilit*” OR TI “functional capa*” OR AB “functional capa*”	344,183
#2	(MM “Absenteeism”) OR (MM “Presenteeism”) OR (MM “Sick Leave”) OR (MM “Job Re-Entry”) OR (MM “Job Performance”) OR (MM “Retirement”) OR (MM “Productivity”) OR (MM “Insurance, Unemployment”) OR (MM “Insurance, Disability+”) OR (MM “Disability Evaluation+”) OR (MM “Work Capacity Evaluation”) OR (MM “Employment Status”) OR (MM “Unemployment”) OR (MM “Self Employment”) OR TI convalescence OR AB convalescence OR TI absenteeism* OR AB absenteeism* OR TI “work absence*” OR AB “work absence*” OR TI “disability absence*” OR AB “disability absence*” OR TI “sickness absence*” OR AB “sickness absence*” OR TI “sick day*” OR AB “sick day*” OR TI “illness day*” OR AB “illness day*” OR TI “work day loss*” OR AB “work day loss* OR TI “work time loss*” OR AB “work time loss*” OR TI “medical leave*” OR AB “medical leave*” OR TI “sick leave*” OR AB “sick leave*” OR TI “sickness leave*” OR AB “sickness leave*” OR TI “disability leave*” OR AB “disability leave*” OR TI presenteeism OR AB presenteeism OR TI “sickness presence” OR AB “sickness presence” OR TI “return to work” OR AB “return to work” OR TI “back to work” OR AB “back to work” OR TI reintegration or AB reintegration OR TI “Reemployment” OR AB “Reemployment” TI “job reentry” OR AB “job reentry” OR TI “work productivit*” OR AB “work productivit*” OR TI “work function*” OR AB “work function*” OR TI “job function*” OR AB “job function*” OR ((TI work OR AB work OR TI job OR AB job OR TI vocational OR AB vocational) AND (TI “Performanc*” OR TI “Efficien*” OR TI “Productiv*” OR “capacity* OR TI disabilit* OR AB disabilit*)) OR TI “employment status” OR AB “employment status” OR TI “work status” OR AB “work status” OR TI “unemployment” OR AB “unemployment” OR TI “unemployed” OR AB “unemployed” OR TI “work abilit*” OR AB “work abilit*” OR TI “workabilit*” OR AB workabilit*” OR TI “work disabilit*” OR AB work disabilit*” OR TI “work inabilit*” OR AB work inabilit*” OR TI “work participation” OR AB “work participation” OR TI “work retention” OR AB “work retention” OR TI “work sustainability” OR AB”work sustainability” OR TI “retirement” OR AB “retirement” OR TI “working hour*”OR AB “working hour*” OR TI “work hour*”OR AB “work hour*” OR TI “work task*” OR AB “work task” OR TI “working task*” OR AB “working task” OR TI “task at work” OR AB “task at work” OR TI “tasks at work” OR AB “tasks at work” OR TI “job task*” OR AB “job task*”	69,142
#1	(MM “Breast Neoplasms+”) OR ((MM “Breast+” OR TI “breast” OR AB “breast”) AND (MM “Neoplasms+” OR TI “neoplas*” OR AB “neoplas*” OR TI “metastas*” OR AB “metastas*” OR TI “cancer*” OR AB “cancer*” OR TI “carcin*” OR AB “carcin*” OR TI “tumour*” OR AB “tumour*” OR TI “tumor*” OR AB “tumor*” OR TI “malig*” OR AB “malig*”))	38,310

## **Appendix 5**: Cochrane Library Search Strategy

**Table:**

ID	Search	Hits	
#1	MeSH descriptor: [Breast Neoplasms] explode all trees	9746	
#2	MeSH descriptor: [Breast] explode all trees	668	
#3	breast:ti,ab,kw	26,635	
#4	#2 or #3		26,646
#5	MeSH descriptor: [Neoplasms] explode all trees		58,274
#6	neoplas* or cancer* or carcin* or tumour* or tumor* or metasta* or malig*:ti,ab,kw (Word variations have been searched)	111,301	
#7	#5 or #6		116,999
#8	#4 and #7	21,704	
#9	#1 or #8		21,704
#10	MeSH descriptor: [Convalescence] explode all trees	132	
#11	MeSH descriptor: [Absenteeism] explode all trees	481	
#12	MeSH descriptor: [Disability Evaluation] explode all trees	2850	
#13	MeSH descriptor: [Sick Leave] explode all trees	485	
#14	MeSH descriptor: [Return to Work] explode all trees		92
#15	MeSH descriptor: [Work Performance] explode all trees	0	
#16	MeSH descriptor: [Work Capacity Evaluation] explode all trees	200	
#17	MeSH descriptor: [Retirement] explode all trees	43	
#18	#10 or #11 or #12 or #13 or #14 or #15 or #16 or #17		3813
#19	convalescen* or absenteeism or work absence* or disability absence* or sickness absence* or sick day* or illness day* or work day loss* or work time loss* or medical leave* or sick leave* or sickness leave* or disability leave* or presenteeism or sickness presence or return-to-work or back- to-work or reintegration or reemployment or job reentry or work productivit* or work function* or work participation or work performance* or performance at work or employment status or work status or unemployment or unemployed or work abilit* or workability or work disabilit* or work inabilit* or work capacit* or work incapacity or work capabilit* or work incapabilit* or work inhibition* or work function* or job function* or work participation or work performanc* or job performanc* or vocational performanc* or performance at work or work productivit* or work efficien* or job efficien* or work retention or work sustainability or retirement* or working hour* or work hour* or work task* or working task* or task at work or tasks at work or job task*:ti,ab,kw (Word variations have been searched)	28,828	
#20	MeSH descriptor: [Efficiency] explode all trees	321	
#21	work* or job*:ti,ab,kw (Word variations have been searched)	35,272	
#22	#20 and #21	166	
#23	#18 or #19 or #22	31,022	
#24	#9 and #23	289	
#25	#24 Publication Year from 2005	213	
#26	MeSH descriptor: [Recovery of Function] explode all trees	3841	
#27	MeSH descriptor: [Disability Evaluation] explode all trees	2850	
#28	MeSH descriptor: [Sickness Impact Profile] explode all trees	520	
#29	MeSH descriptor: [Physical Fitness] explode all trees	2444	
#30	MeSH descriptor: [Movement] explode all trees	23,319	
#31	impairment* or disabilit* or capabilit* or capacit* or impair* or function* or dysfunction* or limitation* or restriction* or physical fitness or movement* or mobilit* or EORTC QLQ or Functional Assessment of Cancer Therapy or SF-36 or functional abilit* or functional capa*:ti,ab,kw (Word variations have been searched)	195,331	
#32	#26 or #27 or #28 or #29 or #30 or #31	206,145	
#34	#9 and #23 and #32	136	
#33	#9 and #23 and #32 Publication Year from 2000	120	
